# Editorial: Major complications in interventional oncology procedures

**DOI:** 10.3389/fradi.2026.1779706

**Published:** 2026-01-28

**Authors:** Alexis Ricoeur, Genti Xhepa

**Affiliations:** 1 Interventional Radiology Unit, University Hospital of Geneva, Geneva, Switzerland; 2 Clinica Di Radiologia, Ente Ospedaliero Cantonale (EOC), Lugano, Switzerland

**Keywords:** chemoembolization, complications, image-guided biopsy, interventional oncology (IO), patient safety, percutaneous intervention, transarterial embolization

Interventional oncology has established itself as a significant component of contemporary cancer treatment. It has garnered the confidence of clinicians and researchers by demonstrating its essential contribution across diverse cancer types. Techniques such as percutaneous ablation, transarterial embolization, chemoembolization, and image-guided biopsies have revolutionized tumor management, underscoring their influence on patient outcomes.

Although these procedures generally possess favorable safety profiles, they nonetheless entail risks of complications that necessitate continuous vigilance. Recognizing that numerous adverse events are not entirely understood underscores the importance of clinician awareness. This emphasizes their responsibility in safeguarding patient safety and maintaining high-quality care. This Research Topic explores these knowledge deficiencies by analyzing complication rates, types, and risk factors associated with interventional oncology procedures. It enables clinicians to make well-informed treatment decisions customized to each patient.

## Technological innovation for improved precision

Effective tumor targeting is imperative for the success of interventional procedures. However, numerous lesions pose challenges to visibility on conventional imaging modalities. Villard et al. conducted an investigation into the utility of fiducial marker placement employing real-time US-CT/MRI fusion imaging to identify poorly visible hepatic tumors prior to percutaneous thermal ablation. In a cohort of 38 cases, 74% of the lesions were completely undetectable on ultrasound. Marker placement was successfully achieved in 79% of the cases. Following fusion imaging, 68% of previously invisible lesions became discernible. Notably, there were no procedure-related complications, demonstrating that technological advancements can enhance both the precision and safety of these interventions.

## Vascular access device complications

Central venous ports facilitate reliable chemotherapy administration; however, they are associated with potential long-term risks. Tanaka et al. present four cases of catheter rupture in central venous ports inserted via the right internal jugular vein. This approach is generally regarded as safer than the subclavian vein approach. All incidents occurred in devices that had been implanted for an average duration of 39 months. Fragment migration to critical regions—including the right atrium, right ventricle, and pulmonary artery—was observed. Notably, all patients remained asymptomatic at the time of rupture detection, and all cases were successfully managed through the use of snare catheters. These findings underscore the importance of regular monitoring for ports that remain indwelling for extended periods.

## Hemorrhagic complications in image-guided biopsy

Zhang et al. conducted a study on bleeding incidents following ultrasound-guided core needle biopsy of infected cervical lymph nodes in a cohort of 643 patients. The overall incidence of bleeding was 23.48%, with internal lymph node hemorrhages accounting for 94% of these cases. Lymph nodes containing pus were associated with a significantly increased risk of bleeding compared to solid lymph nodes (*χ*^2^ = 12.00, *P* = 0.001), and tuberculous lymphadenitis was also linked to a higher bleeding risk relative to common bacterial infections. These findings indicate that preoperative contrast-enhanced ultrasound may serve as a predictive tool for bleeding risk, thereby facilitating the implementation of appropriate preventive measures.

## Sedation-related adverse events

Oxygen desaturation commonly occurs during percutaneous ablation performed under moderate sedation. Wang et al. conducted a prospective randomized trial comparing high-flow nasal cannula oxygen therapy (40 L/min) with low-flow oxygen (6 L/min) in a cohort of 100 patients undergoing radiofrequency ablation. The findings demonstrated that high-flow oxygen significantly decreased the incidence of moderate desaturation (4% vs. 30%; RR 7.5, *P* = 0.0009) without exacerbating any other adverse events. This straightforward approach offers a practical means of enhancing procedural safety.

## Chemoembolization for complicated colorectal cancer

Advanced colorectal cancer can complicate treatment, impacting over 30% of advanced cases. Ding et al. examined transcatheter arterial infusion chemotherapy combined with lipiodol chemoembolization in 54 patients. Clinical success was seen in 83.3% of cases. The objective response rate stood at 66.67%, while the disease control rate was 88.9%. The median obstruction-free survival was 5.0 months, with overall survival at 13.0 months. Importantly, no severe adverse events, such as perforation or major bleeding, were reported, indicating this is a safe option for this difficult-to-treat group.

## Embolization complications: a cautionary case

Boonlorm and Nisityotakul report the initial severe adverse event following transarterial microembolization for a micro arteriovenous fistula in a patient with a chronic venous ulcer. Despite successful embolization, the patient experienced severe leg swelling, erythema, and cellulitis, necessitating extended hospitalization. The authors contend that misdirected embolization, coupled with post-embolization inflammation, contributed to notable skin breakdown. This case underscores the significance of meticulous patient selection and technique, particularly for individuals with pre-existing vascular conditions.

## Multimodal treatment in rare malignancies

Rahul et al. present a case involving a 44-year-old woman with high-grade urothelial carcinoma that transformed into enteric-type adenocarcinoma. This rare variant accounts for only 0.5%–2.0% of bladder cancers. After chemotherapy failed and the tumor progressed, she underwent radical anterior exenteration and received adjuvant radiotherapy. Histopathology showed perineural and lymphovascular invasion. During follow-up, the patient remained disease-free. This example highlights the importance of careful integration of interventional and radiation oncology within comprehensive treatment plans for aggressive tumor types.

## Conclusions

The articles within this Research Topic enhance our comprehension of complications associated with interventional oncology procedures. Key themes encompass technological advancements that improve precision while avoiding increased risks, the necessity for vigilant monitoring of delayed complications, and the identification of patient-specific risk factors. They also emphasize effective management strategies and underscore the significance of meticulous patient selection for emerging techniques. These insights assist clinicians in customizing treatment decisions and in establishing standardized protocols that maximize the optimal balance between risks and benefits. As the field of interventional oncology progresses, continuous documentation of complications remains essential to ensure the safe and effective delivery of minimally invasive cancer therapies.

